# Facilitative Effect of a Generalist Herbivore on the Recovery of a Perennial Alga: Consequences for Persistence at the Edge of Their Geographic Range

**DOI:** 10.1371/journal.pone.0146069

**Published:** 2015-12-30

**Authors:** Moisés A. Aguilera, Nelson Valdivia, Bernardo R. Broitman

**Affiliations:** 1 Centro de Estudios Avanzados en Zonas Áridas (CEAZA), Universidad Católica del Norte, Ossandón 877, Coquimbo, Chile; 2 Instituto de Ciencias Marinas y Limnológicas, Facultad de Ciencias, Universidad Austral de Chile, Campus Isla Teja s/n,Valdivia, Chile; Università di Genova, ITALY

## Abstract

Understanding the impacts of consumers on the abundance, growth rate, recovery and persistence of their resources across their distributional range can shed light on the role of trophic interactions in determining species range shifts. Here, we examined if consumptive effects of the intertidal grazer *Scurria viridula* positively influences the abundance and recovery from disturbances of the alga *Mazzaella laminarioides* at the edge of its geographic distributions in northern-central Chilean rocky shores. Through field experiments conducted at a site in the region where *M*. *laminarioides* overlaps with the polar range edge of *S*. *viridula*, we estimated the effects of grazing on different life stages of *M*. *laminarioides*. We also used long-term abundance surveys conducted across ~700 km of the shore to evaluate co-occurrence patterns of the study species across their range overlap. We found that *S*. *viridula* had positive net effects on *M*. *laminarioides* by increasing its cover and re-growth from perennial basal crusts. Probability of occurrence of *M*. *laminarioides* increased significantly with increasing density of *S*. *viridula* across the range overlap. The negative effect of *S*. *viridula* on the percentage cover of opportunistic green algae—shown to compete for space with corticated algae—suggests that competitive release may be part of the mechanism driving the positive effect of the limpet on the abundance and recovery from disturbance of *M*. *laminarioides*. We suggest that grazer populations contribute to enhance the abundance of *M*. *laminarioides*, facilitating its recolonization and persistence at its distributional range edge. Our study highlights that indirect facilitation can determine the recovery and persistence of a resource at the limit of its distribution, and may well contribute to the ecological mechanisms governing species distributions and range shifts.

## Introduction

Much emphasis has been placed on the importance of biotic interactions and their potential to influence the distribution of species at different spatial and temporal scales [1–5]. Together with environmental constraints, competition, predation, facilitation, and mutualism can determine species’ distributional limits [4–6]. Few studies, however, examine the influence of consumer effects on resource recovery and persistence at both consumer and resource range limit, which can be relevant for species geographic distributions. Theoretical studies suggest that trophic interactions could significantly account for species geographic co-occurrences and range shifts [2,7]. Since range edge populations are characterized by low effective population size and generally low dispersal potential [8], they appear especially prone to local extinction triggered by both abiotic and biotic factors [3,9]. In this way, edge populations of resources could potentially expand or contract depending on variation of environmental barriers to dispersal and the intensity and type of consumption experienced at different life stages; i.e. the magnitude and direction of trophic interactions. This idea is in part related to predictions of the ‘stress-gradient hypothesis’ (SGH) [10], which suggest that the frequency of competitive and facilitative interaction between species pairs vary inversely across gradients of physical stress or ecosystem productivity [11]. Thus, the rate of predation and facilitative interactions at the geographic range of a species is a matter of great interest to understand changes in species distribution in face of global change [12].

Herbivore-plant interactions are one of the most important consumer-resource interactions in terrestrial and aquatic ecosystems, with herbivores having both negative and positive effects on plants from local to biogeographic scales [13–17]. In marine communities, molluscan herbivores can dramatically reduce algal biomass and alter algal distribution patterns and spatial structure [15,16,18–22]. Grazing on competitively superior species or epiphytes can indirectly facilitate recruitment and growth of subordinate macroalgal species at small spatial scales [23,24]. Some marine herbivores can also directly influence vegetative tissue growth through stimulating overcompensation [25], by spore dispersion [26], and addition of nitrogen from excreta [27,28]. Thus, the direction of grazing effects commonly depends on algal life history traits and grazer feeding behavior [29–31]. For example, grazers can have stronger negative effects on ephemeral than calcareous algae, as the latter are resistant to consumption [30] and can benefit from moderate grazing. The magnitude of effects, however, depends on grazer body size and density [14], which usually vary across the geographic range of a species [16]. Given the stressful conditions experienced by algae at their range edge, herbivores could facilitate their persistence [32] and increase the potential of range expansion. Whilst the negative and positive impacts of herbivores on the fitness of their resources have been well documented, their consequences for algal range distribution have been poorly examined (see [14]). This information can be useful to predict the influence of trophic interactions on potential range shifts of resources, given changes in the demography and per capita effects of key consumers across either geographic or physical gradients [33,34].

Overall, at the range edge of grazer and/or algal species, recovery from disturbances and persistence is expected to be sensitive to environmental drivers and variations in grazer–alga interaction strength. On the one hand, it is likely that, at their geographic range limit, herbivores could have moderate or null effects on algal abundance, because suboptimal environmental conditions could negatively affect grazers’ population densities, foraging activity, or both [8,35]. On the other hand, given low densities of algae at their range limit—due for example to low connectivity with source population or because individuals are beyond their physiological tolerance threshold [36]—edge populations can be especially prone to local extinction [37]. In these cases, species-specific grazing on dominant competitors can facilitate the recovery and consequently the persistence of the subordinate algae [23,38].

Along the coast of northern-central Chile, a broad transitional zone is located around 30°S-41°S [39,40]. A 200 km wide section on the northern edge of this area, between 30°S-32°S, is the polar or equatorial edge of the geographic range of several intertidal species. This zone comprises the complete geographic range of the northern–clade of the corticated alga *Mazzaella laminarioides* [41], while the molluscan grazer *Scurria viridula* finds here its polar limit. Thus, the complete geographic range of the northern clade of this alga overlaps with the herbivore across a zone limited to around 200 km, potentially establishing an herbivore-alga interaction system. It has been observed that the northern clade of *M*. *laminarioides* has a strong population structure with signs of recent reduction of population size [41]. In addition, a small population of this species has been observed in a site ca. 120 km northern of its actual range limit. This fragmented distribution and unstable population structure [41] can be caused by discontinuities in physical oceanographic conditions in sea surface temperature (SST) [42], and/or biotic conditions experienced at its range, which has not been previously examined. Previous experimental studies conducted south of the range overlap, where a southern clade of *M*. *laminarioides* is present [41], showed that most species of the intertidal molluscan grazer assemblage can have strong negative effects on early successional algal species [43,44]. In addition, some species of the generalist grazer assemblage can also have positive consumptive effects on the establishment of *M*. *laminarioides* during late succession [44,45]. However, this effect seems to be transient and concentrated on early life stages of the corticated alga species. It is unclear what are the implications of grazing on *M*. *laminarioides* local recovery, persistence and spatial distribution, which might modulate the potential range expansion or contraction of marginal populations of the northern clade of this alga. Because *M*. *laminarioides* has low dispersal potential, its spatial recovery, persistence and distribution might be influenced by *S*. *viridula*, which is the larger and dominant grazer in mid and high intertidal levels in northern Chile [46].

**Fig 1:**
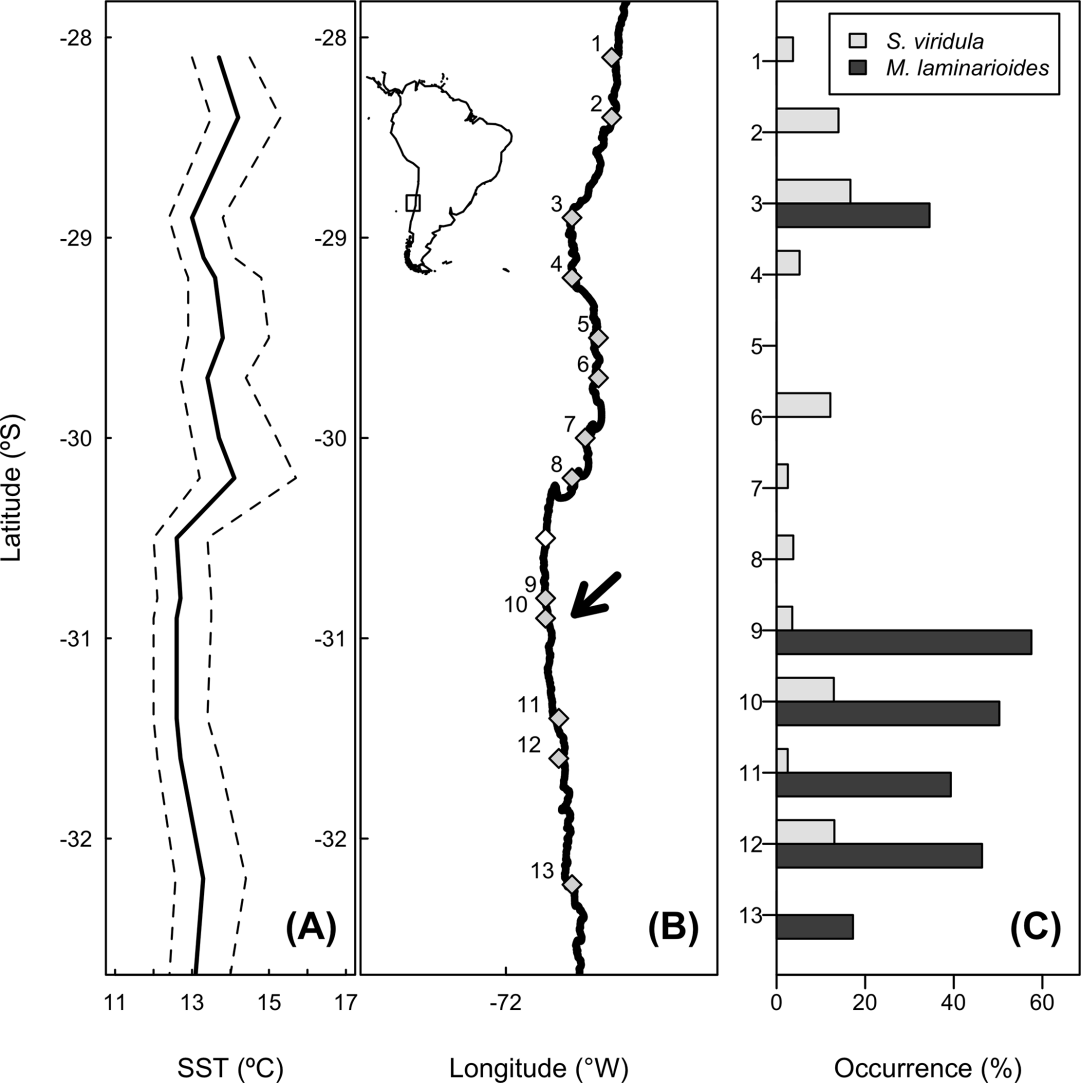
Map of the northern-central coast of Chile showing (a) spatial variation in sea surface temperature (SST) for the 0.25, 0.5, and 0.75 quantiles of the SST distributions recorded at each site for the 2009–2011 period, (b) location of study sites, and (c) percentage of plots where each of the four focal species was recorded at each site. The arrow in panel (b) indicates where manipulative experiments were conducted (site 10, Punta de Talca) and the open diamond indicates the location of a site (Talcaruca) where we only recorded SST. Note that the herbivore and the alga species coexist only at sites 9, 10, 11 and 12 which correspond to the complete range of the northern clade of *M*. *laminarioides* [37].

Using a mixture of intensive field observational surveys (thirteen-year dataset) and manipulative experiments, we examined the effect of the grazer *S*. *viridula* on the recovery, persistence, abundance, and spatial distribution of the northern clade of the late successional algal species *M*. *laminarioides* across the section of coastline where this species overlaps with the herbivore. It has been observed that opportunistic algal species (e.g. *Ulva* spp.) can persist in absence of herbivores outcompeting *M*. *laminarioides* [44,45]. Clumps of *M*. *laminarioides*, as other *Mazzaella* algae species, can recolonize via regrowth from perennial basal crusts and fronds trimmed by disturbances like strong waves, heat stress, and disintegration of fronds by spore release [47]. Thus, we hypothesized that *S*. *viridula* could indirectly facilitate *M*. *laminarioides* growth and abundance by grazing their main algal competitors, or by stimulating direct compensation of vegetative tissue (“facilitation-hypothesis”). Thus, we predicted that across the range overlap the probability of occurrence of *M*. *laminarioides* depends positively of the density of *S*. *viridula* independently of major environmental gradients, such as the latitudinal variation in sea surface temperature (SST) (Fig 1A). Occurrence patterns of the algae could resemble the geographic distribution and small scale density pattern of the herbivore. Alternatively, given the homogeneous genetic population structure of the northern clade of *M*. *laminarioides* [41], settlement and abundance of this species could be curtailed by direct consumption of early developmental stages [44,48], negatively affecting its recovery from, mechanical, disturbances and producing small-scale spatial heterogeneity (“consumption-control hypothesis”). Thus, the abundance of the alga could be negatively correlated with grazer density patterns. We tested these hypotheses using a field experiment, simulating mechanical disturbances through clipping *M*. *laminarioides* fronds and removing the entire alga from the substrate in presence and absence of the focal herbivore. In addition, we assess the main drivers of *M*. *laminarioides* distribution, including the presence of *S*. *viridula*, and the influence of physical factors, using a species distribution model (SDM) fitted to the long-term abundance data.

## Materials and Methods

### Ethics Statement

All invertebrate manipulation in the field and laboratory were conducted according to relevant national and international guidelines. No animals were sacrificed during the experimental study or during the geographic surveys. In addition, the study sites are not privately owner, so that no permits for access were needed. Field activities and experiments did not involve endangered or protected species.

### Study system and focal species

The study region corresponds to the pole-ward endpoint of the range of *S*. *viridula*, and to the range of the northern clade of *M*. *laminarioides* (for simplicity, hereafter referred to as *M*. *laminarioides*) [39–41,49], conforming a ‘range overlap’ spanning about ~200 km (see Fig 1). Along this biogeographic region, *M*. *laminarioides* forms extensive stands and dominates the mid- and high intertidal zones. The opportunistic algae *Ulva rigida*, *U*. *compressa*, and *Pyropia orbicularis* are abundant in high to mid-intertidal levels, interspersed by patches of the crusts *Hildenbrandia lecannellieri* and *Ralfsia* sp. Chthamalid barnacles form extensive patches in mid- to high intertidal levels and the mussel beds characteristic of central-southern Chile are scarce [50]. The herbivore assemblage of the mid-high intertidal shore is characterized by chitons, the keyhole limpet *Fissurella crassa*, and scurrinid limpets like *Scurria zebrina*, *S*. *viridula*, and *S*. *ceciliana* to a lesser extent.

The corticated alga *M*. *laminarioides* has low dispersal potential, is long-lived, and, as other *Mazzaella* species, its recruits may coalesce into dense clumps that persist as a basal crust during stressful periods [47,51]. After damage produced by disturbances like strong waves, heat stress, and disintegration of fronds by spore release, individual clumps of this alga recolonize via regrowth from perennial basal crusts and trimmed fronds [47]. The northern clade of *M*. *laminarioides* shows signs of a recent bottleneck, following a reduction of effective population size [41].

*Scurria viridula* is among the largest generalist molluscan grazer of the assemblage [46,48] of the region north of the range overlap (mean ± Standard Error of the Mean of maximum body length: 2.62 ± 0.15 cm), reaching high densities and frequencies of occurrence at the northern limit of the range overlap (11.9 ± 1.34 ind. m^-2^, Fig 1B and 1C). According to their radular scraping capabilities it can be considered an efficient grazer, capable of removing macroalgal spores and small plantlets at very high rates [29]. Large densities of this species north of the range overlap suggest that this grazer species might have large population-level consumptive effects on algal communities. Previous studies suggest that *S*. *viridula* has expanded its geographic distribution by around 100 km [52], but recent observations suggest this expansion is larger (M. A. Aguilera & B. R. Broitman unpublished results). There is no available information, however, concerning potential algal range shift. Similarly, the influence of subtle abiotic gradients (e.g. sea surface temperature, **Figure A in S1 File**) and biotic factors on the distributional limit of these species is still unclear.

### Geographic monitoring of the grazer-alga co-occurrences and sea surface temperatures (SST)

In order to determine co-occurrence distributional patterns (i.e. presence/absence measures) of the focal species across and beyond the range overlap, we used long-term abundance datasets (i.e. 1998–2011) obtained from a monitoring scheme conducted at fifteen sites spanning ~700 km of coastline from 28°S to 33.5°S (Fig 1). At each site, the presence of *M*. *laminarioides* and the density of *S*. *viridula* was recorded using 20 50 × 50 cm quadrats (divided in 25 fields), haphazardly positioned along transects about 15 m in length on two platforms in mid intertidal levels. Detailed explanations of sampling methods and schedules are published elsewhere [39,53]. Although we aimed to test whether grazer occurrences determine the alga occurrence pattern, the causality of the relationship can well be opposite (i.e. the algal occurrence determines the grazer density). We recorded SST by means of temperature dataloggers (Hobo, Onset) moored at ~1-meter depth (i.e. below zero level) at each site to represent the nature of the environmental conditions in the range overlap. The temperature dataloggers were monitored every 3–4 months between 2009 and 2013.

### Small-scale spatial distribution and association

To determine if grazer and algal species were randomly, aggregated, or uniformly distributed over rocky platforms, and to characterize their patterns of spatial association, we recorded *S*. *viridula* density and *M*. *laminarioides* cover at different sites across the geographic range overlap (Fig 1B). We counted all individuals of *S*. *viridula* and recorded the percentage cover of *M*. *laminarioides* using 36 to 50 30 × 30 cm contiguous quadrats positioned along two 10 to 15 m-transects parallel to the shoreline at each study site. The sampling unit size considered has been shown to be appropriate to detect significant spatial variation in abundance of *Scurria* limpets [46], as most of them forage about 12 cm around their home scars [54]. Similarly, this quadrat size allows for a representative site-scale quantitative characterization of the intertidal community, including foliose algae like *M*. *laminarioides*. The sampling was conducted between October 2010 and July 2011 in the mid-intertidal zone at three sites located in the range overlap (i.e. Limarí, Punta de Talca, and Huentelauquén; sites 9, 10, and 11, respectively, in Fig 1B and 1C).

### Grazer-alga interaction strength: field experiments

To determine the consumptive effects of *S*. *viridula* on *M*. *laminarioides* abundance, persistence, and distribution at small spatial scales, we conducted an enclosure/exclusion experiment in the mid-intertidal level in the locality of Punta de Talca (30.9°S, 71.6°W, Site 10 and arrow in Fig 1B), which is located at the northern edge of the range overlap. According to the scraping feeding mode of scurrinid limpets, we assumed that consumptive effects of *S*. *viridula* on *M*. *laminarioides* concentrate on spores, basal crusts, and small plants [48]. In addition, we considered the consumptive effects of this grazer on short trimmed fronds of *M*. *laminarioides*, because the fronds of this alga partially disintegrate after releasing the carpospores and can be completely removed after consumption by browser herbivores (e.g. *Fissurella crassa*, [44,55]) or trimmed by fish (authors’ personal observations, Escobar and Navarrete unpublished data). Hence, as grazing by *S*. *viridula* may affect the rate of frond recovery (from basal crusts) and growth rate, we evaluated the effect of *S*. *viridula* grazing on different life stages of *M*. *laminarioides*. We selected 15 25 × 25 cm areas (see **Figure A in S1 File**) and in the center of each we left two clumps (4–6 cm diameter) of adult *M*. *laminarioides* distant ~6 cm from each other. One clump was trimmed at the basal portion leaving small erect thalli (1.5 cm, green in **Figure A in S1 File**), which mimicked the height of senescent fronds after spore release. For the second clump, fronds were completely removed leaving only the basal crustose thalli from where fronds can re-grow (brown patches in **Figure A in S1 File**). The rock surface around clumps was scraped clean with drill-mounted brushes and manual chisels, removing all organisms including encrusting algal fragments. This last procedure was intended to mimic conditions during early community succession. It is worth noting that in intertidal habitats release of primary substrate is a frequent process generated by carnivores, grazers, and physical disturbance [52]. Experiments started during the season of vegetative growth and spore production of *M*. *laminarioides* (early summer to autumn), and finished before spore release and frond senescence (mid-winter to early spring). Each experimental area was randomly assigned to one of the three following treatments (n = 5): (a) Enclosure of two *S*. *viridula* (3.4 ± 0.16 cm shell length), according to local natural densities of this species in the range overlap [31], (b) exclusion of benthic grazers, where all herbivores were removed from the plots, and (c) control”open access” areas where all herbivores were allowed to enter and graze. Each plot allocated to treatment (a) or (b) was fenced with a 6 cm-high wire mesh deployed around the experimental area (see **Figure A in S1 File**, for exclusion/enclosure effectiveness), while those allocated to treatment (c) were marked with stainless-steel bolts (see **Figure A in S1 File**).

Manipulative field experiments similar to our study have used partial fences to control the potential artefact of fences on algal growth or grazer consumption. We conducted a pilot study to test the applicability/usefulness of this treatment in our study system (**Figure A in S1 File**). Our pilot study suggested that partial fences can affect experimental *M*. *laminarioides* clumps, because these procedural control were quickly removed by strong waves that are common in our study site (**Figure A in S1 File**). Thus, we did not include this treatment in our design because of its potential to alter algal growth and establishment. Although we did not include a ‘fence effect’ in our treatment design, a recent, temporally more intensive study conducted at the same site, and with comparable treatment design, suggested that fences can have minimal, and non-significant, confounding effects on algal recruitment and abundance (**Figure A in S1 File**). In addition, previous studies conducted in this system suggest that small height of the fences considered in the experiment (6 cm) do not enhance or impede algae recruitment and/or grazing rate compared with partial fences or open areas [44,56]. Additionally, we stringed atop of each fence a thin transparent nylon fishing line (0.1mm) to reduce the potential impact of fish grazing. Only two nylon lines were stringed across sides to create a grid that was wide enough (about 225 cm^2^) to avoid shading inside the experimental area. Previous studies have found no effect or artefact of this procedure (e.g. neither on algae growth nor recruitment), which is intended to reduce fish grazing inside experimental areas in fenced plots (enclosures and exclusion treatments) when compared with completely open areas [44].

**Fig 2:**
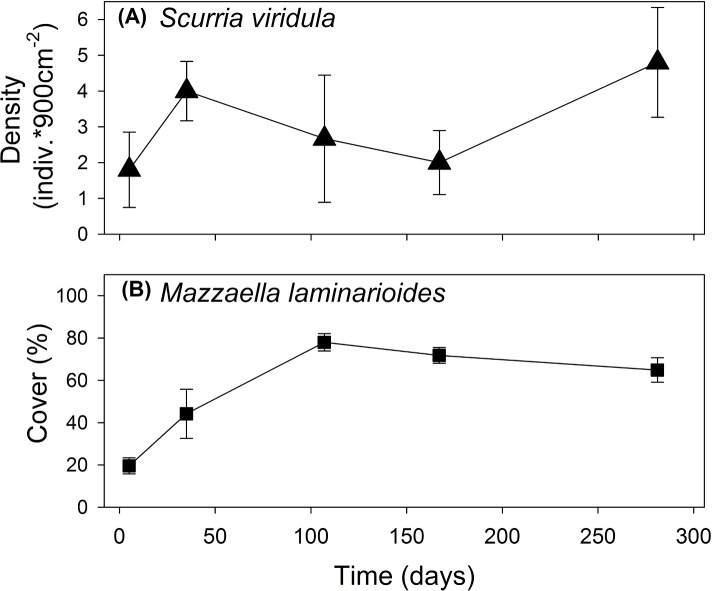
Predicted probability (± 95% CI) of occurrence of the corticated algae *Mazzaella laminarioides* determined by density of the grazer *Scurria viridula* found at four sites located within the range overlap estimated through logistic regressions.

In order to estimate grazing intensity in experimental “open access areas” and to follow algal settlement, we measured every two months the density of herbivores and both adult and juvenile *M*. *laminarioides* in the experimental platforms (Fig 2A). The percentage cover of bare space in enclosure and control was considered as a direct measure of foraging rate of the focal grazer species in each experimental set compared with exclusions (grazer-free areas). Thus, this measure can be considered as a net effect of the focal grazer integrating positive indirect effect on *M*. *laminarioides* through foraging on competitors. Monthly from December 2009 to May 2010, we monitored the percentage cover of all macrobenthic (> 3 cm) sessile organisms occurring in each experimental area by means of 25 × 25 cm quadrats (81 uniformly spaced intersection points). At each sampling date, the whole benthic community in each plot was photographed with a high-resolution digital camera and percentage cover estimations were conducted in the laboratory. Monthly, we also measured the length of all *M*. *laminarioides* fronds re-grown from trimmed clumps. The area of each basal crust, and the number of fronds regrowth from them were measured at the end of the experiments.

#### Statistical analyses

Using information from the long-term monitoring program across different sites, we conducted multiple logistic regression analyses, with site latitude, maximal local (0.75 quantile) SST, and densities of *S*. *viridula* as explanatory variables, and presence/absence of *M*. *laminarioides* as dependent variable. Maximum likelihood was used to estimate model parameters. Before the analyses, we computed for each explanatory variable its variance inflation factor (VIF) in order to detect collinearity. The term with highest VIF was sequentially removed until all remaining terms showed VIF < 3 [54]. Akaike and Bayesian information criteria (AIC and BIC respectively) were used to find the best fit to the probability of occurrence of *M*. *laminarioides*. We compared all possible regression models using the meifly package in the R environment version 3.0 (R Development Core Team 2013). The model with the lowest AIC and BIC values was selected as the best fit (see **Table A in S1 File**).

The small-scale spatial structure (cm to meters) of *S*. *viridula* and *M*. *laminarioides* abundance data was analyzed using spatial correlograms with Moran’s *I* [57]. We determined the statistical significance (α = 0.05) by bootstrapping our observations [58]. The significance at each lag was estimated with the distribution of autocorrelation coefficients obtained by randomly re-sampling the data set and recalculating the coefficients 1000 times. A global autocorrelation test was conducted by checking whether each lag contained a least one significant correlation after probabilities were adjusted using a Bonferroni correction for multiple tests (α’ = 0.05/number of distance classes). Interspecific spatial association of the focal grazer and algal abundance at small-scales (cm to meters) were estimated through Pearson’s correlations (r) between the grazer density and alga percent cover. Significance was calculated through a t-test in which the degrees of freedom were corrected according to the degree of autocorrelation (Moran’s *I*) between data sets (quadrats at the same distance classes) in the contiguous quadrat sampling data [59]. Correlations at each study site were calculated on averaged abundance data.

For the manipulative experiment, changes in percentage cover of ulvoids and bare rock, in addition to canopy cover and growth rates of trimmed *M*. *laminarioides* clumps were analyzed using one-way repeated measures analysis of variance (RM-ANOVA), using time as the repeated factor. Trimmed fronds and crusts of *M*. *laminarioides* clumps were analyzed separately. The former started to grow three months before the end of the experiment, thus we considered only this time interval for analyses of algal growth and canopy cover. All data were log-transformed to improve variance homogeneity. The Hyund-Feldt correction was used to adjust degrees of freedom when data did not meet sphericity assumptions for univariate tests [60]. In the case of significant effects among treatment differences (between subjects), we used the following planned contrasts: (1) to evaluate effects of all herbivores (total herbivory) we compared the control versus exclusion, (2) to evaluate the effects of enclosed *S*. *viridula* versus those of other herbivores, we compared enclosures versus control and (3) to evaluate the effect of *S*. *viridula* in absence of other herbivores we compared enclosure versus exclusions. Dunn-Šidák correction was used to adjust significance levels for the multiple contrasts performed. As we did not include a procedural control in our experiment (see arguments above and in **Figure A in S1 File**), comparisons between fenced areas and open plots (controls), which test for the effect of all herbivores of the system, should be considered with caution.

Differences in re-grown frond density among treatments were tested with a one-way ANOVA using the temporal average frond density of each *M*. *laminarioides* crust in each plot. Data were log-transformed in order to meet the assumption of homogeneity of variance, which was graphically explored by means of residuals-vs.-fits plots. In the case of significant effects, we used planned contrasts as described above.

In order to determine the direction and magnitude of grazing effects on algae and to provide comparable information we estimated per capita interaction strength in field experiments. Within plots, colonization of ephemeral algae started a few days after rock clearance and reached an established stage after 18–20 weeks, about the colonization phase of early successional algal species in the region. Hence, we estimated the consumptive effect of natural densities of *S*. *viridula* on dominant ephemeral algae and on bare rock cover, considering their average cover pooled for the first twenty weeks of the study. We also estimated grazer effects on *M*. *laminarioides* frond cover.

To quantify the interaction strength (per capita effects) considering natural densities of *S*. *viridula* we used the “Dynamic Index” (DI) suggested for resources with positive exponential growth, such as early-succession species and ephemeral algae (e.g.[35,61]). The index was calculated as: **DI = (ln (CovEN/CovEX))/N**×**t,** where CovEN is the mean specific algal or bare rock cover in the *S*. *viridula* enclosures, CovEX is the mean algal cover in the grazer exclusions, **N** is the density of *S*. *viridula* in the experimental plots and **t** is the elapsed time of experiments, in this case in weeks. We also computed DI × natural density of *S*. *viridula* observed within and north of the range overlap in order to estimate population effects [62] of this grazer. It is worth noting that although this measure can provide useful information about potential grazer population-level effects, it assumes linear *per capita* effects across a range of densities of the grazer (see [62,63]). Thus, results of these analyses must be interpreted with caution [63]. An average population effect ≤ 1 (100% of plants removed by herbivores) indicates either total prevention of algal recruitment or production of 1 m^2^ of bare rock per week. This allowed us to evaluate potential impact of the *S*. *viridula* on *M*. *laminarioides*, when natural densities in the range overlap zone resemble those found north of its range edge. Confidence intervals (95%) for effect estimates were obtained through a bootstrapping procedure [58].

## Results

### Geographic co-occurrences at the range overlap

Coastal ocean temperature across the region showed a persistent and localized decrease in SST from ~14.5°C at 28°S to 12.8°C at 30.5°S (Fig 1A) over the period of 13 years (i.e. 1998–2011) which is consistent with the distributional pattern and northern range edge of *M*. *laminarioides*. We also observed a drop in SST at a single study site located ~29° S (Los Burros), where an isolated local population of *M*. *laminarioides* is located over 120 km equatorward of the previously reported edge of the range (Fig 1A).

Quadrat surveys showed that both *S*. *viridula* and *M*. *laminarioides* had higher incidences (i.e. number of times a species was found at a site over the period considered) across the range overlap when compared with sites north (see Fig 1C). For the case of *M*. *laminarioides*, Akaike and Bayesian information criteria (AIC and BIC, respectively) were smaller for the model that included *S*. *viridula* density, latitude, and maximal SST as explanatory variables (Table 1). According to the selected model, the density of *S*. *viridula* contributed significantly to the prediction of *M*. *laminarioides* probability of occurrence (**Table A in S1 File**). The odds ratio for *S*. *viridula* was estimated as 1.12 with 95% CI ranging from 1.05 to 1.20 (Fig 2, and Table A in S1 File), indicating that for one unit of increase in *S*. *viridula* density, a quadrat had ca. 1.12 chance of having *M*. *laminarioides* compared to not having the alga, an increase in the odds of having *M*. *laminarioides* of ca. 10%. Maximal SST was also an important contribution to *M*. *laminarioides* occurrence across the range considered (Table 1, and **Table A in S1 File**).

**Table 1:** Model selection according to Maximum Likelihood (logL) parameter estimation, Akaike and Bayesian Information Criteria (AIC and BIC, respectively) for the occurrence of the corticated alga *M*. *laminarioides*. The model with the lowest information criteria is in bold. Latitude (Lat), maximal SST (SSTmax), and density of the grazer *S*. *viridula* (Scu) were included in the full model as explanatory variables. Minimal and median SST were excluded from the full model due to high variance inflation ratios. Coefficient for the intercept is presented before each other terms of the model.

logL	AIC	BIC	Model
**-605.29**	**1218.59**	**1240.22**	**21.85 + 0.060 Lat—1.721** SST_max_ **+ 0.110 Scu**
-612.07	1230.15	1246.37	23.35 + 0.034 Lat—1.764 SST_max_
-673.92	1353.85	1370.07	23.05–1.673 SST + 0.108 Scu
-675.50	1354.99	1365.81	24.02–1.736 SST
-836.19	1678.39	1694.61	6.04–0.188 Lat + 0.121 Scu
-851.81	1707.62	1718.43	7.16–0.221 Lat
-1018.38	2040.75	2051.56	0.20 + 0.127 Scu

### Small-scale distribution and association

**Fig 3:**
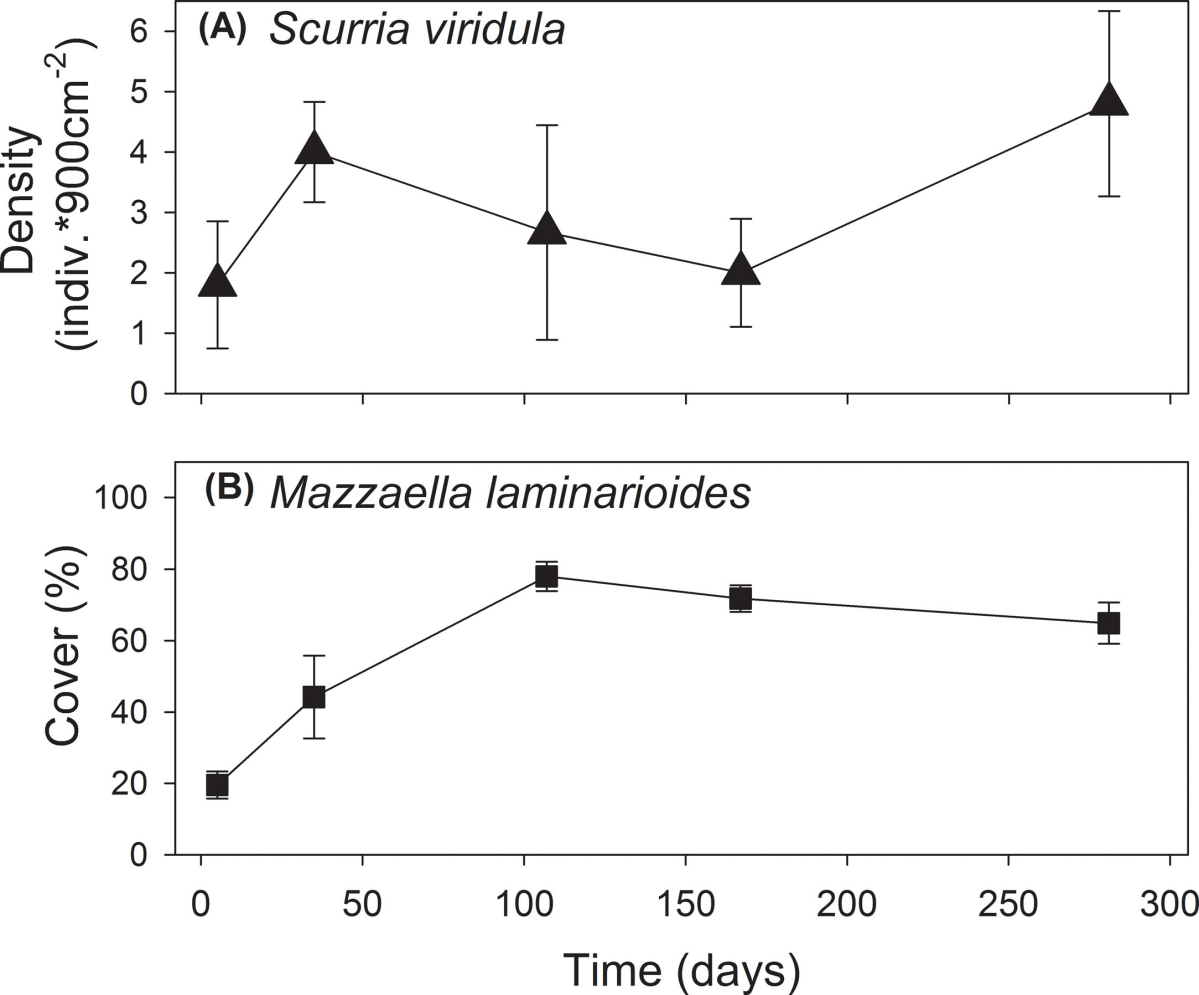
Average abundance (±SE) of (a) the grazer *Scurria viridula* and (b) the corticated alga *Mazzaella laminarioides* recorded in the experimental platforms at Punta Talca, located at the core of the range overlap of the two species.

Small-scale spatial surveys (cm to meters) showed that *S*. *viridula* density remained relatively constant through time and along the shoreline at the experimental study site (Fig 3A). Densities of this grazer were similar across the range overlap, with average densities (± SEM) of 2.36 ± 0.68, 3.05 ± 0.58, and 2.17 ± 0.74 ind. 900cm^-2^ at Limarí, Punta Talca, and Huentelauquén, respectively. Average canopy cover of *M*. *laminarioides* was around 20% at the start of the study (summer) in mid-intertidal levels in the study site, stabilizing around 100 days from the beginning of the study at ~70% (Fig 3B). Average percentage cover measured between late summer and early autumn across the range overlap was 32.12 ± 6.06, 55.7± 10.67, and 32.82 ± 6.09% 900 cm^-2^ in Limarí, Punta Talca, and Huentelauquén, respectively. The analysis of intra-specific spatial structure at small scales (cm to meters) showed that the grazer species was randomly distributed at all sites (see **Table B in S1 File**). Similarly, the spatial distribution of *M*. *laminarioides* was random at Limarí, but showed a more aggregated pattern at Punta Talca and Huentelauquén according to significant autocorrelation estimates at the lower distance classes (i.e. lag 0, about 30–90 cm, see **Table B in S1 File**). According to our broad-scale analysis of species occurrences, abundances of *S*. *viridula* and *M*. *laminarioides* were positive and significantly correlated at the scale of quadrats (30 × 30 cm) in two sites within the range overlap (Limarí and Huentelauquén, Table 2).

**Table 2:** Summary of Pearson spatial correlation (r) between densities of S. viridula and cover (%) of M. laminarioides at three sites located in the range overlap. Modified t-tests were performed to determine significant differences in the herbivore-alga spatial correlation. Degrees of freedom and P-values were adjusted by presence of spatial autocorrelation in the data set (Dutilleul`s correction).

	r	P
Limarí	0.301	0.039*
Punta de Talca	0.068	0.841
Huentelauquén	0.229	0.0137*

Significance is indicated as P < 0.05*

### Grazer-alga interaction strengths

**Fig 4:**
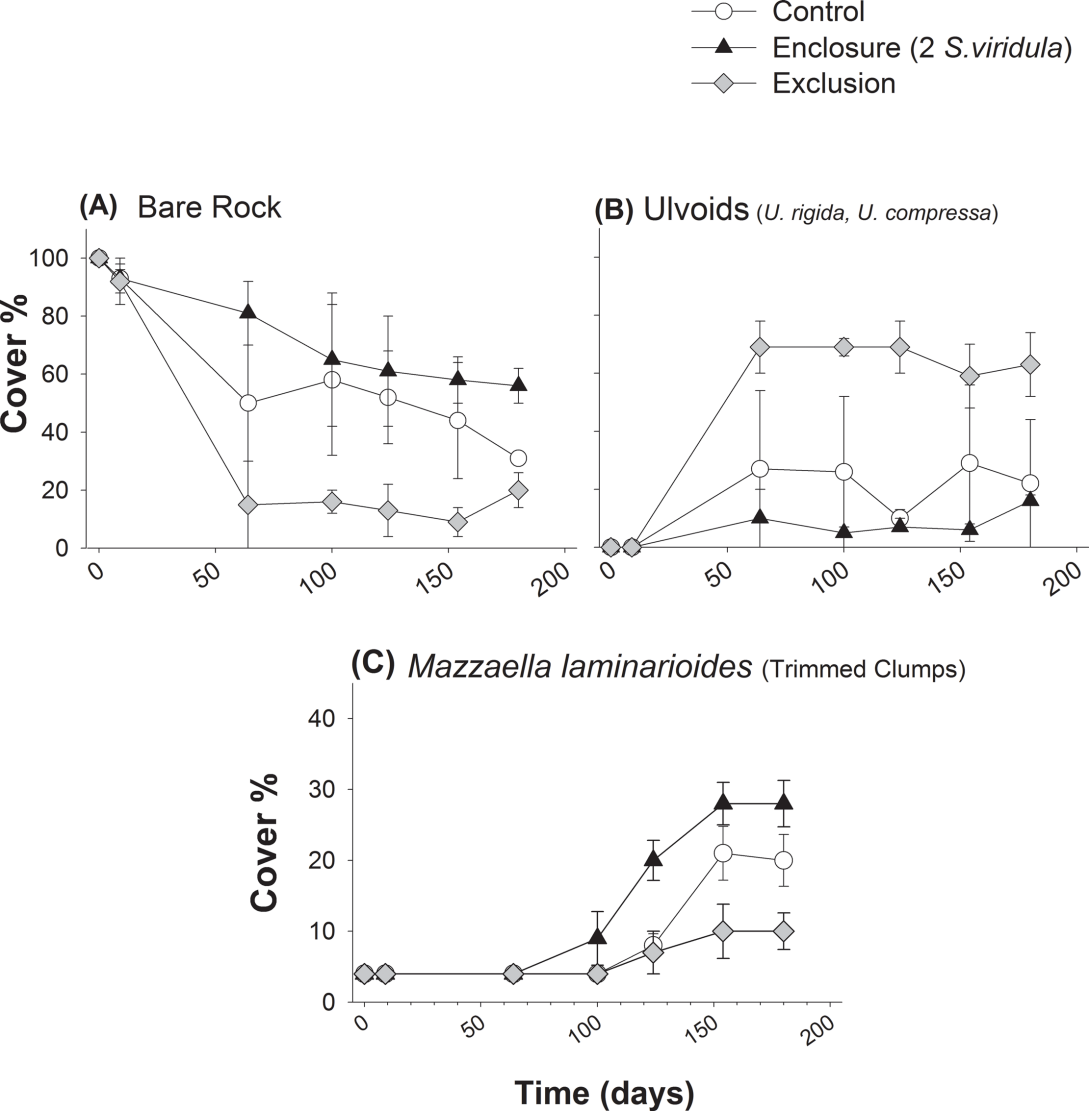
Main ecological changes recorded inside experimental plots in the field experiments, corresponding to enclosure of the grazers *Scurria viridula*, exclusion of all grazers and ‘open areas’ (control). Average percentage cover (± SE) of (a) bare substrate, (b) green opportunistic ulvoid algae and (c) the corticated alga *Mazzaella laminarioides*.

Grazing by *S*. *viridula* in enclosure plots significantly increased bare rock cover by reducing the abundance of fast-growing ulvoid algae (*Ulva rigida* and *Ulva compressa*) throughout the study when compared with exclusion plots (Fig 4A and 4B, and see **Table C in S1 File**). Effects on bare rock and ulvoid algae cover were variable through time (i.e. significant Time*Treatment effects; **Table C in S1 File**). Grazing effects of enclosed *S*. *viridula* on bare rock and ulvoids were not statistically different from those observed in control free-access plots (see planned contrast in **Table C in S1 File**). These results suggest that grazing effects on bare rock and ulvoids in free-access plots were mostly due to *S*. *viridula* grazing. Trimmed clumps of *M*. *laminarioides* inside experimental areas started growing after 100 days from the beginning of the experiment (see Fig 4C). In presence of *S*. *viridula*, percentage canopy cover of trimmed plants of *M*. *laminarioides* was significantly higher than in exclusion areas (see Fig 4C, and **Table C in S1 File**), suggesting that the presence of the grazer benefited the regrowth of the trimmed fronds. We observed a slightly, albeit not significantly, higher canopy cover in control areas compared with exclusion areas (Fig 4C, see planned contrasts in **Table C in S1 File**).

**Fig 5:**
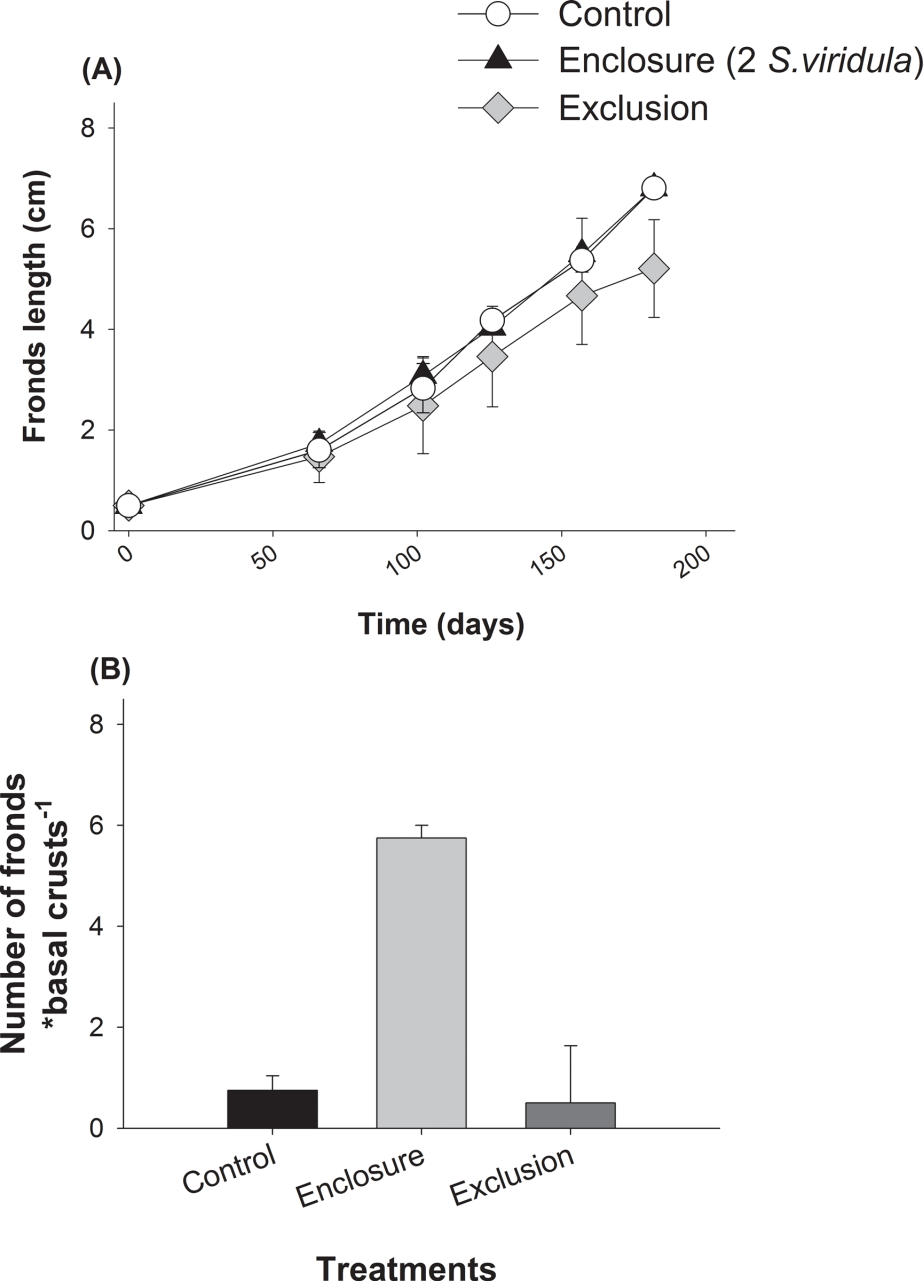
Performance of the corticated alga *Mazzaella laminarioides* in the presence and complete absence of *Scurria viridula* inside experimental plots. (a) Mean (± SEM) fronds length (cm) of plants trimmed at the start of the experiment and (b) average number of fronds (± SEM) regrown from basal non-calcareous crusts created inside experimental plots.

Average length of trimmed fronds of *M*. *laminarioides* clumps was not significantly different among treatments after ~200 days from the onset of the experiment (see Fig 5A and **Table D in S1 File**). After 5 months of study, small fronds of ~2 cm in length of *M*. *laminarioides* grew from basal crusts generated at the start of the experiments. At the end of the study, the density of small fronds growing from these crusts was significantly higher in *S*. *viridula* enclosures than in exclusion and control plots (Table 3, Fig 5B). In contrast, frond density was not significantly different between grazer exclusion and control plots (planned contrasts: Table 3).

**Table 3:** One-way ANOVA on density of frond re-growth from basal crust of the alga *M*. *laminarioides* presents inside the experimental treatments. P-values were adjusted using Dunn-Sidák correction for multiple test.

Source	Df	MS	F	P
Treatment	2	0.4199	7.69	0.0115*
Error	9	0.0549		
Planned Constrasts				
Control vs Enclosure	1	0.5404	9.84	0.0356*
Control vs Exclusion	1	0.0113	0.21	0.9624
Enclosure vs Exclusion	1	0.7082	12.89	0.0173*

P < 0.05*.

**Fig 6:**
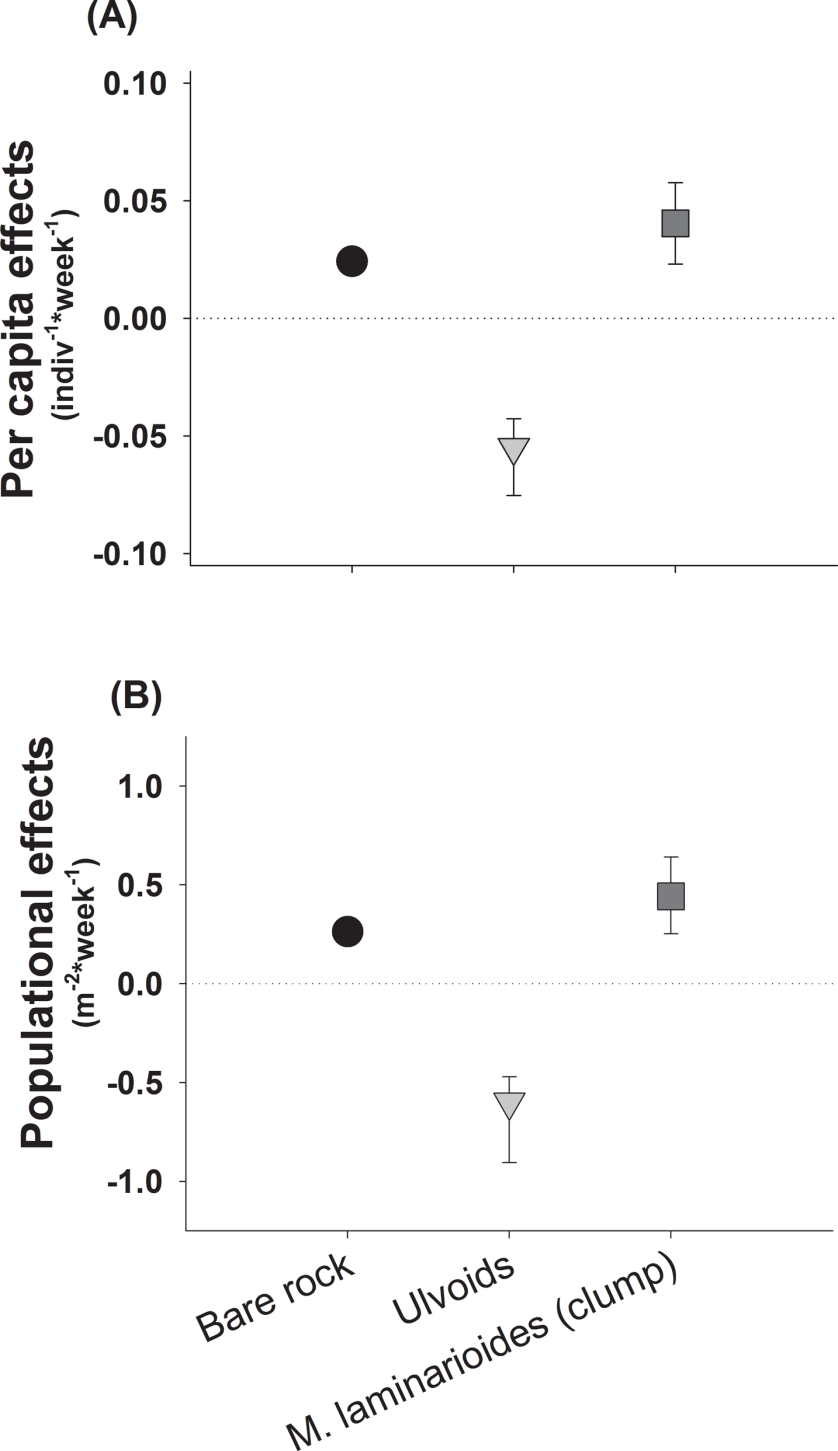
Strength and direction of the interaction between the grazer *S*. *viridula* and the alga *M*. *laminarioides*, measured as the grazer capacity to influence the recruitment of algae and bare rock production during early succession, and the recovery *M*. *laminarioides* fronds (i.e. Average per capita (a) and population effects (b)). Bars are 95% confidence intervals estimated through a bootstrapping procedure.

*S*. *viridula* had clear per capita effects on both bare rock availability and ulvoid cover of the early successional community (Fig 6). Significant negative average per capita effects on ulvoids were mirrored by positive, albeit moderate, effects on bare rock availability (see Fig 6A). Enhanced growth of trimmed fronds of *M*. *laminarioides* inside *S*. *viridula* enclosures took place during the final experimental stage (see Fig 4A). In general, we found that *S*. *viridula* had positive effects on canopy cover of trimmed plants of *M*. *laminarioides* (Fig 6A). Estimation of population effects of *S*. *viridula* considered natural densities of this species recorded at the study site and northern the range overlap. Expected average population effects of *S*. *viridula* showed that this grazer was able to remove around 60% of ulvoids (spores and plantlets) per m^2^ per week (Fig 6B). According to the positive per capita effects observed in our field experiments, natural populations of *S*. *viridula* enhanced *M*. *laminarioides* canopy cover by about 44% per m^2^ per week in the range overlap zone (Fig 6B). In the locality of Los Burros, distant about 120 km northern its range edge, a population of *M*. *laminarioides* still persists (cover = 34.1 ± 7.18%, Fig 1C) and population effect of *S*. *viridula* were estimated to enhance in canopy cover in 15.8%. In sites about 200 km northern the ‘range overlap’ (i.e. Carrizal Bajo) where densities of *S*. *viridula* are high but *M*. *laminarioides* is yet absent, it is expected that *S*. *viridula* might enhance *M*. *laminarioides* canopy cover by about 38%. Meanwhile considering the densities recorded for this grazer southern the range overlap (i.e. about 250 km, in Las Cruces), it is expected that this species might contribute to enhance *M*. *laminarioides* cover and frond regrowth by about 5% per m^2^ per week.

## Discussion

At its range limit, the grazer *S*. *viridula* showed strong effects on populations of *M*. *laminarioides* by facilitating frond re-growth after mechanical disturbance. In line with our hypothesis of positive effects, *S*. *viridula* negatively affected the percentage cover of opportunistic green algae, which are suggested to affect the growth of corticated algae. Accordingly, we found a positive spatial correlation, at different scales, between the herbivore and algal species throughout the range where both overlap in their distribution.

We found a consumer-resource dynamic that was likely established through both indirect and direct effects in the species range overlap [64,65]. On the one hand, the negative grazing effects on opportunistic green algae could have resulted in an indirect positive effect on *M*. *laminarioides* through competitive release. Rapid nutrient uptake and high growth rates confer opportunistic algae the ability to outcompete perennial, slow-growing macroalgae in the absence of external disturbances [66]. Competition for light and nutrients between opportunistic and perennial algae is intense, but most grazers prefer to feed on early life stages of opportunistic algae [38,66]. It has been also shown that removal of opportunistic algae can facilitate the recovery of algal canopies with more complex morphological forms especially in systems where disturbances are more intense [67]. Thus, as suggested by the ‘stress-gradient hypothesis’[11], some combinations of life history traits and stress factors can switch ecological interactions from negative (e.g. consumptive control) to positive in plant-herbivore interactions [32]. These evidence suggests the hypothesis that, through selective grazing on spores and recruits of competitively dominant opportunistic ulvoids (mainly *U*. *rigida* and *U*. *compressa*), *S*. *viridula* could indirectly benefit the recovery and abundance of *M*. *laminarioides*. On the other hand, corticated algae with a crustose phase or with a persistent basal crust formed by tightly arranged cells, like *M*. *laminarioides*, may tolerate and benefit from intense grazing through compensatory growth or uptake of nutrients from herbivore’s excretions [27,38]. Such direct positive effects of grazers on algal persistence have been shown in other marine systems (e.g. coral reefs, [68]). For example, stimulation of meristematic tissue by grazing, the chiton *Choneplax lata* increases growth of the coralline algae *Porolithon pachydermus* [68]. Since perennating basal crusts or holdfast are key structures of recolonization of different *Mazzaella* species [47], we could propose a second, competing hypothesis predicting that moderate grazing by *S*. *viridula* on these structures could contribute to *M*. *laminarioides* recovery through stimulating the re-growth of fronds. In agreement with this hypothesis, we found that at the end of our experiment the cover of *M*. *laminarioides* frond (of the single trimmed clump) was about 30% in *S*. *viridula* enclosures compared with 18% in control (open areas). In natural stands of *M*. *laminarioides* sampled with the quadrat-sampling protocol in the same study site, we observed that *M*. *laminarioides* covered 71.8% of the substratum in autumn. On the same sampling date, we observed that approximately two or three entire clumps (8cm across) of *M*. *laminarioides* were present within each sampling quadrat (900 cm^2^), with each clump accounting about 25% to 36% to the total *M*. *laminarioides* cover. This suggests that the ‘recovery potential’ of *M*. *laminarioides*, measured as the capacity of frond re-growth from trimmed clumps, was indeed enhanced by *S*. *viridula* grazing. It should be noted that as we did not include an ‘intact’ *M*. *laminarioides’*clump treatment in our experiments, algal recovery to a reference natural state in presence of the herbivore needs to be considered with caution. Therefore, further manipulative work is needed to disentangle the processes (e.g. competitive release and compensatory growth) generating positive algal responses in correlation to grazing. Climate change factors, such as eutrophication and ocean acidification, have been shown to benefit the proliferation of ephemeral and foliose algae; according to recent experimental work conducted elsewhere [69] and our results, grazing activity can exert a firm control on ephemeral algae and thus act as a compensatory mechanism that might counteract the impacts of disturbances on natural communities.

Significant positive spatial associations between grazer density and *M*. *laminarioides* cover was detected in two sites, corresponding with the patch structure of the alga. Grazer distribution, however, was random at all study sites precluding any inference about potential causal relationships between grazer and algal distribution at small scales. Spatial structure could be related to processes like patchy recruitment patterns and/or post-settlement mortality. Meanwhile, a lack of spatial structure in species distribution is commonly attributed to either the effects of random spatial processes, or that samples were collected over unappropriated spatial scales [57]. In this context, intertidal grazer distributions can change from gregarious when resting to either random or uniform while foraging [54]. Likely, our sampling protocol only included the foraging phase of the grazer which could have a more gregarious spatial patterns during the resting period. Nonetheless, previous studies suggest that the spatial scale considered in our study is appropriate to determine abundance of the study species [46,54]. In addition, competitive interaction (interference) with other grazers could also affect the distribution pattern of *S*. *viridula* (e.g.[70]), but no studies have been conducted to test these hypothesis.

In central Chile, previous field and laboratory experiments have identified grazers, such as *Chiton granosus* and *Scurria araucana*, as able to enhance recruitment and abundance of *M*. *laminarioides* [44,45]. These species show similar grazing strategies as *S*. *viridula*, removing spores and plantlets by a “scraping-grazing” foraging mode. Together, these species could have an additive, or even redundant, contribution to the maintenance of *M*. *laminarioides* across the range of this alga. In central Chile, coastal localities where nutrient supply is locally higher show increased negative effects of the grazer assemblage on ephemeral algae, and increased growth rates of corticated alga like *M*. *laminarioides* [43]. Our study site, Punta Talca, is located near a persistent upwelling center [42]. As expected from the local drop in SST observed at this location, growth and recovery potential of *M*. *laminarioides* is expected to be high due to high nutrient concentrations associated with coastal upwelling activity [43]. According to the low fronds re-growth rates and high bare rock cover observed in control areas of our field experiments, we suggest that other functionally different and abundant herbivores like the keyhole limpet *Fissurella crassa*, the pulmonate *Siphonaria lessoni* and fish have a strong negative effect on abundance and recovery of *M*. *laminarioides* through browsing on its fronds (e.g. [44]). Therefore, the functionally diverse local herbivore assemblage can impact different life stages of the corticated algae through differential grazing modes thus producing contrasting effects on algae distribution. Hence, both competitive interaction of *M*. *laminarioides* with opportunistic fast-growing algae like ulvoids and browsing by other grazers, such as large keyhole limpets, could prevent re-regrowth and control abundance of this alga in natural conditions. The lack of procedural controls in our experiment may hinder clear-cut interpretations of the differences between open (all herbivores effects) and fenced areas (exclusion, *S*. *viridula* enclosure). Nonetheless, a recent study conducted in this system showed no confounding effect of fences neither on algal recruitment nor abundances (see **Figure A in S1 File**, and see also [56] for low shore habitat). Thus, we interpret that the differences in *M*. *laminarioides* growth and abundance observed in our experiments were driven by the presence/absence of the focal herbivore species and not through experimental artefacts of the exclusion/enclosure method utilized. It is worth noting that appropriate procedural controls are necessary in field exclusion experiments, and these treatments should be included in experimental studies, either as a previous pilot study and/or directly in the final design.

Theoretical studies have shown the importance of considering biotic interactions as part of species distribution models (e.g. [2,6,71]). Biotic interactions can interact with abiotic factors like desiccation or wave stress to determine intertidal species distributions [34]. The significant contribution of maximal SST to our species distribution model (SDM) reinforces the role of abiotic factors on biogeographic patterns of intertidal species [36,72]. For example, changes in nearshore oceanographic conditions can influence the connectivity and dispersion of intertidal organisms (e.g.[37,50,73]). Similarly, SST can be negatively correlated with nutrient supply in coastal habitats, which in turn influences the herbivore-alga dynamic [43,74]. Our SDM showed that including the grazer *S*. *viridula* in the logistic model improved the estimation of *M*. *laminarioides* occurrence probability over SST alone. Notwithstanding, we recognize the limitations of our sampling methods and the difficulty to infer causality from the significant relationship between grazer density and *M*. *laminarioides* occurrence. Recent evidence suggests that *S*. *viridula* has expanded its range about 200 km south of its historical range edge, despite no evident changes in environmental drivers like SST having been detected (but see [75]). However, no information is available about potential changes in the distribution of *M*. *laminarioides* (expansion or contraction of its range) regarding changes in environmental trends ([75], and see below).Considering the low local densities of this grazer found in the newly colonized range (e.g. Las Cruces), the expected population effects on the southern clade of *M*. *laminarioides* is still low compared with its effects estimated for the northern clade in the study site (an increase of about 5.2% of algal cover versus 38% estimated in the study site). Thus, the potential for *M*. *laminarioides* to expand its range edge northward might be mediated by limited dispersal [73,76], the ability of spores to grow up under higher heat stress [34,77], and also by facilitative grazing effect on the re-growth and persistence of this alga (this study). Thus, net grazing effects on re-growth from disturbances and persistence of algae may have an influence on algal range shifts when physical conditions open a window of opportunities for colonization in new geographic ranges. Transplants of *M*. *laminarioides* beyond its range edge are biologically risky because of accidental dispersal of propagules, so we did not perform experiments north of the range overlap zone. We found *M*. *laminarioides* (fronds cover = 34.1% ± 7.18) in Los Burros, located about 120 km northern its established range edge. This isolated population could be a relic after past northern expansion of *M*. *laminarioides* by relaxation in barriers to dispersal (i.e. upwelling fronts). Demographic and phylogeographic studies provide cursory support for this interpretations showing that edge populations have reduced genetic diversity and are highly structured [41]. Nonetheless, as we observed in our study, persistence of the isolated population of *M*. *laminarioides* at Los Burros is strongly associated to low SST, and to a lesser extent by indirect facilitation of *S*. *viridula*. In this context, our results suggest that *S*. *viridula* enhances the recovery of *M*. *laminarioides* following disturbances by about 16%. We also estimated that about 230 km north of its current equatorial range edge, where *M*. *laminarioides* is not present (i.e. Carrizal Bajo), densities of *S*. *viridula* are equivalent to those presents at Punta Talca. Thus, densities of this grazer are expected to be high enough to influence the recovery of *M*. *laminarioides* to the north of its range edge. Since no effects were observed on the establishment of this alga (i.e. spore settlement-germination), physical factors (e.g. limiting spore dispersion and heat stress [34,73]) seem to be critical for its establishment at this site. In this way, changes in physical factors like sea and air temperature wind and/or acidification, at the magnitudes predicted by global change models [78], could constrain the distributional range of *M*. *laminarioides* while an increase in nutrients concentration by upwelling intensification and grazer indirect facilitation ([43], and this study) could compensate this effect at its range edge [69]. Hence, an interplay between negative control of spore dispersion/settlement by physical stress and positive influences of grazing on recovery and persistence seem thus to be important for maintaining *M*. *laminarioides* geographic distribution.

In conclusion, trophic interactions can have a key role as post-settlement processes, operating at small spatial scales and probably influencing species recovery from disturbances and population persistence at broader scales. Grazing effects by *S*. *viridula* on different life stages of *M*. *laminarioides* improve their ability to recover from disturbances likely via enhancement of recolonization from perennial basal crusts, ameliorating interspecific competition, or a combination of both processes. Other herbivores, however, have negative effects on the re-growth of fronds of *M*. *laminarioides* and can thus control its abundance and distribution in natural conditions. Our findings suggest that while physical barriers to dispersal are relevant to determining species range distribution and establishment via limitation of spore dispersion [79], variation in the intensity and magnitude of biotic interactions can influence the ability of species to persist or collapse at the edge of their geographic range. Such local processes, in turn, can be critical for species to further expand or contract their range after physical barriers are relaxed by environmental variation.

## Supporting Information

S1 File Details of the field experimental procedures conducted for the herbivore-alga pair, and tables with statistical results. Preliminary experiments and enclosure/exclusion treatment design of field experiments utilized to test the effect of *Scurria viridula* on the ability of algae to colonize and regrowth from trimmed clumps and basal crusts (**Figure A**). Maximum likelihood estimates of model parameters and model selection for *Scurria-Mazzaella* interaction and Sea surface temperature (SST) (**Table A**). Summary of spatial autocorrelation analysis at small (cm to meters) spatial scales for the herbivore and the algae species (**Table B**). Summary results from repeated measures ANOVA of a) bare rock and b) ulvoids (i.e. *Ulva compressa*, *U*. *rigida*) found in cleared areas of the experimental plots in the *Scurria-Mazzaella* field experiment (**Table C**). Repeated measures ANOVA of a) percent canopy cover and b) frond length of trimmed clumps of *Mazzaella laminarioides* (**Table D**).
(DOC)
