# Correction: Facilitative Effect of a Generalist Herbivore on the Recovery of a Perennial Alga: Consequences for Persistence at the Edge of Their Geographic Range

**DOI:** 10.1371/journal.pone.0148303

**Published:** 2016-02-04

**Authors:** Moisés A. Aguilera, Nelson Valdivia, Bernardo R. Broitman

Fig 2 appears incorrectly in the published article. Please see the corrected version here.

**Fig 2:**
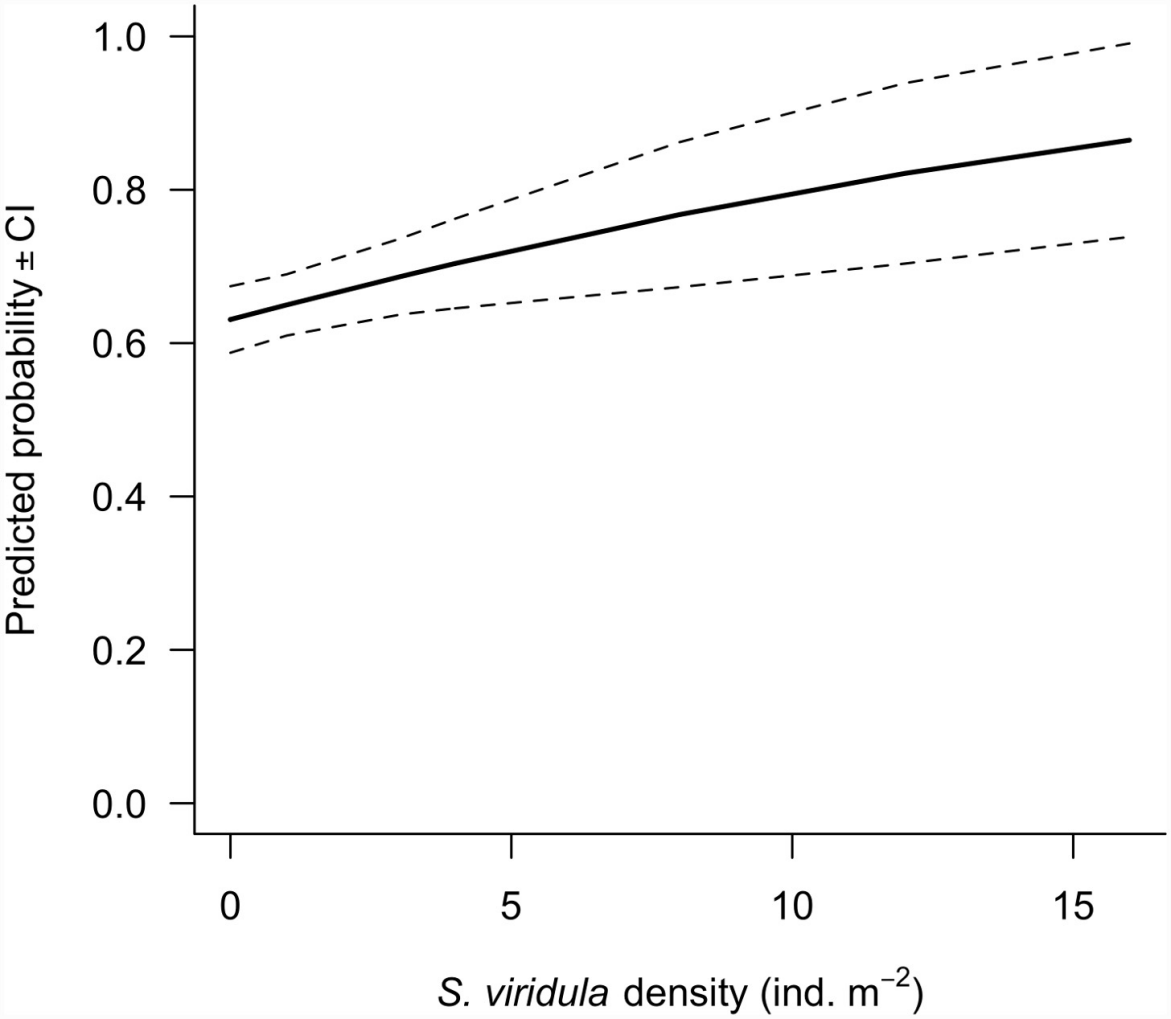
Predicted probability (± 95% CI) of occurrence of the corticated algae *Mazzaella laminarioides* determined by density of the grazer *Scurria viridula* found at four sites located within the range overlap estimated through logistic regressions.
