# Supplementary material for: Facilitative Effect of a Generalist Herbivore on the Recovery of a Perennial Alga: Consequences for Persistence at the Edge of Their Geographic Range
Source: PLoS One. 2015 Dec 30;10(12):e0146069. doi: 10.1371/journal.pone.0146069 (PMC4696856; doi:10.1371/journal.pone.0146069)
Supplement: S1 File — Preliminary experiments and enclosure/exclusion treatment design of field experiments utilized to test the effect of Scurria viridula on the ability of algae to colonize and regrowth from trimmed clumps and basal crusts (Figure A). Maximum likelihood estimates of model parameters and model selection for Scurria-Mazzaella interaction and Sea surface temperature (SST) (Table A). Summary of spatial autocorrelation analysis at small (cm to meters) spatial scales for the herbivore and the algae species (Table B). Summary results from repeated measures ANOVA of a) bare rock and b) ulvoids (i.e. Ulva compressa, U. rigida) found in cleared areas of the experimental plots in the Scurria-Mazzaella field experiment (Table C). Repeated measures ANOVA of a) percent canopy cover and b) frond length of trimmed clumps of Mazzaella laminarioides (Table D). (DOC) [file pone.0146069.s001.doc]

Supporting information

**Scheme of experimental methods**

**and**

**Tables with statistical results**

**Facilitative effect of a generalist herbivore on the recovery of a perennial alga: consequences for persistence at the edge of their geographic range**

Moisés A. Aguilera^1^**^*^**^#a^, Nelson Valdivia^2^ and Bernardo R. Broitman^1^

^1^Centro de Estudios Avanzados en Zonas Áridas (CEAZA), Universidad Católica del Norte, Ossandón 877, Coquimbo, Chile

^2^Instituto de Ciencias Marinas y Limnológicas, Facultad de Ciencias, Universidad Austral de Chile, Campus Isla Teja s/n,Valdivia, Chile

^#a^Current Address: Departamento de Biología Marina, Facultad de Ciencias del Mar, Universidad Católica del Norte, Larrondo 1281, Coquimbo, Chile

**Figure A. Details of the field experimental procedures conducted for the herbivore-alga pair.**

**Exclusion/enclosure method and effectiveness**

The experiments consisted of a) *S. viridula* enclosures, b) benthic grazer exclusion and a c) control (open areas) treatment (Fig. A). In c) we evaluated the potential effect of all different benthic herbivores presents in the study site. We enclosed/excluded benthic grazers using stainless steel fences (6 cm high, 7 mm mesh opening) fastened to the rock with stainless-steel bolts, an effective field experimental procedure to reduce benthic grazer migration into experimental units [1]. Since *S. viridula* is the main grazer species in mid-high shore elevation, on these shores we assumed that experimental grazer enclosures resembled natural conditions of grazing pressure. Artefacts caused by fences on algae colonization were tested previously by comparing a “partial fence” treatment (procedural control) with a control (open areas), and grazer “complete” fence treatment. These preliminary assays were conducted in mid and low intertidal levels in a wave exposed platform in the study site [see also 2, for details]. After one month in the field, partial fences were easily removed by waves, producing mechanical disturbances on the emergent community that colonized the plots. Thus, this treatment was not further considered in the further experimental design.

Because we did not include a “fence effect” treatment in this experiment, we were unable to determine if fence increased algal cover (ephemeral algae, *M. laminarioides*) in exclusion areas compared with the “open access” areas. Nevertheless, results obtained in the low-shore assay showed no significant effect of fences in the abundance of different algal groups [2]. In this context, as we focuses on the variation in abundance of *M. laminarioides* in presence and absence of *S. viridula* (enclosed inside fences), so that total exclusion areas were assumed as “referential condition” (true control) with the same conditions as enclosures. Therefore, we expected to capture most of the general patterns of grazing effects.

A recent study conducted in the same site and intertidal level, and using the same plot size and with comparable treatment design (limpet enclosure/exclusion), showed that algal cover was similar between control (open areas) and partial fences used as procedural control (Aguilera et al. unpublished). In that study, after 13 month of experiments, percent cover of the main algal groups showed no statistical differences between these treatments (% cover differences; control v/s procedural control, Tukey’s HDS pairwise test after one-way ANOVA: ulvoids = 12.9, P = 0.099; *Hildenbrandia* = 2.13, P = 0.383; *M. laminarioides* = 0.671, P = 0.927; *Pyropia* sp. = 4.28, P = 0.946; *Ulvella* sp. = 8.22, P = 0.167). These results suggest that fences had no significant effects on algal recruitment and abundance. Nonetheless, percent cover of bare rock was higher in partial fences compared with control areas (% cover differences, Tukey’s HDS test: 20.71, P = 0.00013). Despite partial fences were maintained until the end of this experiment, and replaced frequently, we observed the same dislodged effect of partial fences as we recorded in our pilot study presented above.

It is worth noting that a partial barrier as an enclosure/exclusion procedural control has been considered inadequate for estimating artefacts associated with exclusion treatments [3,4]. Despite “cageless” methods to exclude benthic grazers as copper paint have been used as alternative to fencing [5,6], these methods are only partially effective to exclude other molluscan grazers (e.g. keyhole limpets [1,6]) compared with fencing the experimental plots.

Since other grazer and browser herbivores are also abundant in the study site like *Siphonaria lessoni*, *Chiton granosus* and the keyhole limpet *Fissurella crassa*, we expected that the joint effect of these species in the control plots (Fig. Ac) were concentrated on trimmed fronds, basal crusts and spore colonization.

Only two enclosed *S. viridula* individual had to be replaced from an enclosure treatment during the study. We monthly removed other grazers found inside enclosure/exclusion plots in the experiments such as *Scurria* spp. and *Siphonaria* *lessoni* recruits (2-3 individual per plot >5mm length). In general, the number of other grazers, e.g. *Chiton granosus*, removed per month from plots was low (<2) compared with observed densities of the study species (*S. viridula*: 11.9 ± 1.34 ind. m^-2^) in the experiments.

25

cm

25

cm

5.0

cm

25

cm

25

cm

6.0

cm

**a)**

*Scurria*

*viridula*

enclosure

**b)**

Benthic grazer exclusion

**c)**

Control (

‘

Open access

’

)

**Figure A.** Experimental design of field experiments utilized to test the effect of *Scurria viridula* on the ability of algae to colonize and regrowth from trimmed clumps and basal crusts in the mid-high intertidal community at Punta Talca, range overlap of the algae and the herbivore species. Rock was scraped clean at the beginning of the experiments and algae allowed to colonize and grow. Adult plants of the corticated alga *Mazzaella laminarioides* (northern clade) were trimmed (green) and scraped to leave basal crusts (brown) at the start of the experiments.

**References**

1. Aguilera MA, Navarrete SA. Functional identity and functional structure change through succession in a rocky intertidal marine herbivore assemblage. Ecology. 2012;93: 75–89. Available: http://www.ncbi.nlm.nih.gov/pubmed/22486089

2. Aguilera MA, Valdivia N, Broitman BR. Herbivore-alga interaction strength influences spatial heterogeneity in a kelp- dominated intertidal community. PLoS ONE. 2015;10: e0137287. doi:10.1371/journal.pone.0137287

3. Johnson LE. Potential and peril of field experimentation: The use of copper to manipulate molluscan herbivores. Journal of Experimental Marine Biology and Ecology. 1992;160: 251–262. doi:10.1016/0022-0981(92)90241-2

4. Benedetti-Cecchi L, Cinelli F. Confounding in field experiments: direct and indirect effects of artifacts due to the manipulation of limpets and macroalgae. Journal of Experimental Marine Biology and Ecology. 1997;209: 171–184.

5. Range P, Chapman MG, Underwood AJ. Field experiments with “cageless” methods to manipulate grazing gastropods on intertidal rocky shores. Journal of Experimental Marine Biology and Ecology. 2008;365: 23–30. doi:10.1016/j.jembe.2008.07.031

6. Aguilera MA, Navarrete SA. Effects of Chiton granosus (Frembly, 1827) and other molluscan grazers on algal succession in wave exposed mid-intertidal rocky shores of central Chile. Journal of Experimental Marine Biology and Ecology. 2007;349: 84–98. doi:10.1016/j.jembe.2007.05.002

7. Nielsen KJ, Navarrete SA. Mesoscale regulation comes from the bottom-up: intertidal interactions between consumers and upwelling. Ecology Letters. 2004;7: 31–41. doi:10.1046/j.1461-0248.2003.00542.x

**Table A.**  **Maximum likelihood estimates of model parameters for *Scurria-Mazzaella* interaction and Sea surface temperature (SST).** Estimates for the relationship between presence of *Mazzaella laminarioides* (i.e. the odds that the alga occurred relative to its absence) and the density of *Scurria viridula* on a 0.25-cm^2^ plot, maximum sea surface temperature (SST_max_), and the latitude. We selected the best fit by analysing Akaike and Bayesian Information Criteria (AIC and BIC respectively).

|  | Estimate | SE | z-value | P | Odd Ratio | 95% CI | |
| --- | --- | --- | --- | --- | --- | --- | --- |
| (Intercept) | 21.852 | 3.958 | 5.521 | <0.001 |  |  |  |
| *Scurria viridula* density | 0.110 | 0.034 | 3.182 | 0.001 | 1.116 | 1.047 | 1.199 |
| SST_max_ | -1.721 | 0.263 | -6.549 | <0.001 | 0.179 | 0.106 | 0.297 |
| South latitude | 0.060 | 0.100 | 0.598 | 0.550 | 1.062 | 0.872 | 1.294 |

**Table B. Summary of spatial autocorrelation analysis at small (cm to meters) spatial scales for the herbivore and the algae species.** Spatial autocorrelation analysis (Moran`s *I*) at lag 0 (around 30-90 cm) conducted on the density of the grazer *S. viridula* and the percentage cover of the alga *M. laminarioides* at three intertidal rocky shores*.* Significance was estimated after bootstrapping (1000 times) and Bonferroni correction. See text for details. P < 0.05*.

|  |  | Limarí | Punta de Talca | Huentelauquén |
| --- | --- | --- | --- | --- |
| *Mazzaella laminarioides* |  | 0.3617  0.0217* | 0.1847  0.1950 | 0.3108  0.0162* |
| *Scurria viridula* |  | 0.0010  0.2268 | -0.0972  0.8590 | 0.1013  0.0669 |
|  |  |  |  |  |
|  |  |  |  |  |

**Table C.** **Summary results from repeated measures ANOVA of field experiments.** Repeated measures ANOVA of a) bare rock and b) ulvoids (i.e. *Ulva compressa*, *U. rigida*) found in cleared areas of the experimental plots in the *Scurria-Mazzaella* field experiment. P-values were adjusted using Dunn-Sidák correction. P < 0.05*, p < 0.01**.

| Source | df | MS | F | P |
| --- | --- | --- | --- | --- |
| **a) Bare rock**  *Between subject* |  |  |  |  |
| Treatment | 2 | 5.2973 | 13.09 | 0.002** |
| Error | 9 | 0.4050 |  |  |
| Planned Constrasts |  |  |  |  |
| Control vs. Enclosure | 1 | 0.5124 | 1.27 | 0.6416 |
| Control vs. Exclusion | 1 | 5.7214 | 14.13 | 0.0134* |
| Enclosure vs. Exclusion | 1 | 9.6582 | 23.86 | 0.0027** |
| *Within Subjects* |  |  |  |  |
| Time | 5 | 0.9209 | 5.29 | 0.0007** |
| Time*Treatment | 10 | 0.3900 | 2.24 | 0.0300* |
| Error (Time) | 45 | 0.1740 |  |  |
| **b) Ulvoids**  *Between subject* |  |  |  |  |
| Treatment | 2 | 5.4226 | 4.73 | 0.0395* |
| Error | 9 | 1.1468 |  |  |
| Planned Constrasts |  |  |  |  |
| Control vs. Enclosure | 1 | 0.00002 | 0.01 | 0.9971 |
| Control vs. Exclusion | 1 | 8.1224 | 7.08 | 0.0260* |
| Enclosure vs. Exclusion | 1 | 8.1454 | 7.10 | 0.0258* |
| *Within Subjects* |  |  |  |  |
| Time | 5 | 2.6677 | 20.42 | <0.0001** |
| Time*Treatment | 10 | 0.3298 | 2.52 | 0.0225 |
| Error (Time) | 45 | 0.1306 |  |  |

**Table D**. Repeated measures ANOVA of a) percent canopy cover and b) frond growth out of trimmed clumps of *Mazzaella laminarioides*, recorded during the last 3 months of field experiments. P-values were adjusted using Dunn-Sidák correction. P < 0.05*, P < 0.01**.

| Source | Df | MS | F | P |
| --- | --- | --- | --- | --- |
| **a)** **Canopy cover**  *Between subject* |  |  |  |  |
| Treatment | 2 | 1.1650 | 4.97 | 0.0371* |
| Error | 9 | 0.2343 |  |  |
| Planned Constrasts |  |  |  |  |
| Control vs. Enclosure | 1 | 0.0728 | 0.31 | 0.09315 |
| Control vs. Exclusion | 1 | 1.3598 | 5.80 | 0.1135 |
| Enclsoure vs. Exclusion | 1 | 2.0621 | 8.80 | 0.0443* |
| *Within Subjects* |  |  |  |  |
| Time | 2 | 0.3036 | 4.17 | 0.0545 |
| Time*Treatment | 4 | 0.1093 | 1.50 | 0.2639 |
| Error (Time) | 18 | 0.0728 |  |  |
| **b)** **Fronds length**  *Between subject* |  |  |  |  |
| Treatment | 2 | 4.0770 | 0.96 | 0.4196 |
| Error | 9 | 4.2560 |  |  |
| *Within Subjects* |  |  |  |  |
| Time | 2 | 17.1756 | 102.14 | <0.0001** |
| Time*Treatment | 4 | 0.3792 | 2.26 | 0.1034 |
| Error (Time) | 18 | 0.1682 |  |  |
